# Surface-Fabrication of Fluorescent Hydroxyapatite for Cancer Cell Imaging and Bio-Printing Applications

**DOI:** 10.3390/bios12060419

**Published:** 2022-06-15

**Authors:** Weimin Wan, Ziqi Li, Xi Wang, Fei Tian, Jian Yang

**Affiliations:** 1 State Key Laboratory of Component-Based Chinese Medicine, Tianjin University of Traditional Chinese Medicine, Tianjin 301617, China; wwm2019@stu.tjutcm.edu.cn (W.W.); lzq0350@stu.tjutcm.edu.cn (Z.L.); wangxi2019@stu.tjutcm.edu.cn (X.W.); tianfei@tjutcm.edu.cn (F.T.); 2 Institute of Traditional Chinese Medicine, Tianjin University of Traditional Chinese Medicine, Tianjin 301617, China; 3 Tianjin Key Laboratory of Phytochemistry and Pharmaceutical Analysis, Tianjin University of Traditional Chinese Medicine, Tianjin 301617, China

**Keywords:** hydroxyapatite, polyethyleneimine, riboflavin sodium phosphate, cell affinity, cell imaging, drug delivery

## Abstract

Hydroxyapatite (HAP) materials are widely applied as biomedical materials due to their stable performance, low cost, good biocompatibility and biodegradability. Here, a green, fast and efficient strategy was designed to construct a fluorescent nanosystem for cell imaging and drug delivery based on polyethyleneimine (PEI) and functionalized HAP via simple physical adsorption. First, HAP nanorods were functionalized with riboflavin sodium phosphate (HE) to provide them with fluorescence properties based on ligand-exchange process. Next, PEI was attached on the surface of HE-functionalized HAP (HAP-HE@PEI) via electrostatic attraction. The fluorescent HAP-HE@PEI nanosystem could be rapidly taken up by NIH-3T3 fibroblast cells and successfully applied to for cell imaging. Additionally, doxorubicin hydrochloride (DOX) containing HAP-HE@PEI with high loading capacity was prepared, and in-vitro release results show that the maximum release of DOX at pH 5.4 (31.83%) was significantly higher than that at pH 7.2 (9.90%), which can be used as a drug delivery tool for cancer therapy. Finally, HAP-HE@PEI as the 3D inkjet printing ink were printed with GelMA hydrogel, showing a great biocompatible property for 3D cell culture of RAW 264.7 macrophage cells. Altogether, because of the enhanced affinity with the cell membrane of HAP-HE@PEI, this green, fast and efficient strategy may provide a prospective candidate for bio-imaging, drug delivery and bio-printing.

## 1. Introduction

Multifunctional nanoparticles are of great interest for new applications such as cell imaging, drug delivery and tumor therapy [1,2,3,4,5]. Increasing surface chemistry strategies are used to establish these nanosystems via surface modification, such as polymer surface coating and small molecule surface grafting [6,7,8]. Among them, small molecule surface modification is one of the most commonly used methods, which has some advantages including fast and mild preparation process, excellent biocompatibility and superior fluorescence characteristics [9,10,11].

Hydroxyapatite (HAP) has attracted a great interest in biomedical fields due to its high stability, low cost, excellent biocompatibility and biodegradability [12,13,14,15]. The surface-functionalized HAP gives it many new properties, and the doping of rare earth ions is an efficient and typical surface modification to make it capable for cell imaging [16,17,18]. For example, He et al. successfully prepared Gd^3+^-doped monodisperse hydroxyapatite by hydrothermal method, which can be used for cell imaging in HeLa cells [19]. Hui et al. grafted an amphiphilic polymer with the hydrophobic end to the surface of a fluorinated Lu3^+^-doped HAP via hydrophobic interactions, which was successfully applied to cell imaging in A549 and Hela cells [20]. Zhou et al. completed the hydrophilic and hydrophobic transition by coating ethanolamine phosphate on the surface of nano-fluorine-doped HAP, and further coupled with quantum dots (QDs) to achieve the cell imaging in HepG2 and Hela cells [21]. However, rare earth materials are expensive and difficult to obtain, and the poor biocompatibility of QDs severely limits their application after modified with HAP. Therefore, the development of efficient and economical HAP surface modification methods is still urgently needed.

Riboflavin sodium phosphate (HE) is an inexpensive soluble small molecule with fabulous fluorescence properties, which has potential application prospects in the field of cell imaging. It has been reported that HE can be grafted on the surface of HAP through a single-ligand-exchange reaction to enable HAP acquire fluorescent properties [22]. However, the HE-grafted HAP may not be suitable for cell imaging because the HE molecules cannot enhance the HAP uptake by living cells. It is significant that nanoparticles for cell imaging mainly rely on the target uptake processes (i.e., endocytosis) by target cells [23,24,25]. Furthermore, with negative charge on the out-surface of the cell membrane, positively surface-charged nanoparticles can be attracted to the cells according to the principle of electrostatic interaction [26,27]. Thus, converting the surface charge of the nanoparticles into a positive charge is one of the effective ways to enhance their affinity with the cell membrane. Therefore, it is of great research significance to further develop rational modification methods to enhance the cell membrane affinity of HAP-HE.

Adsorption of a layer of cationic polymer on the surface of HAP-HE is an ideal modification to increase positive surface charge with the advantages of being green, mild and fast [28]. Polyethyleneimine (PEI) is a commonly used commercial cationic polymer with considerable adhesion properties. Thereby, modification of the cationic layer on the surface of HAP-HE can be easily achieved by using the PEI that has a strong adhesion ability.

Three-dimensional (3D) cell culture systems using cell scaffolds are currently under development. They not only better simulate the in vivo environment, but also have the advantages of intuition and controllability of cell culture. Application of 3D bio-printing strategies in these 3D models provides soluble gradients as the printing ink for cell patterning. However, it is still limited due to the lack of suitable materials for biological linkage [29]. Gel materials encapsulating live cells with good biocompatibility to form biological links are urgently required. To date, GelMA, deriving from a photoreactive gelatin derivative with the common arginyl-glycyl-aspartic acid (RGD) and matrix metalloproteinase (MMP) sequences, has shown promising advantages [30]. The introduction of GelMA during 3D bioprinting can both protect cells during extrusion and provide biological signals to embedded cells as the structures mature during culture [31]. Here, we initially explored the imaging effect of fluorescent nanomaterials (HAP-HE@PEI) on macrophage RAW264.7 cultured in GelMA.

**Scheme 1:**
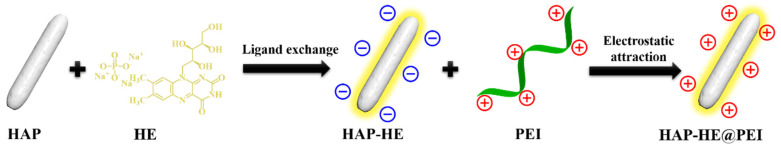
Preparation process of HAP-HE@PEI with positively charged surface.

In this study, we designed a fast, simple and mild establishment of multifunctional for the preparation of HAP-HE@PEI that is constructed by HE-modified HAP (HE-HAP) via a ligand exchange process to render it fluorescence property, and then wrapped PEI onto the surface of HE-HAP via electrostatic attraction to enhance cell membrane affinity of HE-HAP due to its high positive surface charge (Scheme 1). We further characterized encapsulation efficiency of doxorubicin (DOX) and pH-dependent release characteristics by HAP-HE@PEI. Moreover, we demonstrated the cell imaging capabilities of HAP-HE@PEI by showing its stable and high-intensity fluorescence accumulation in cytoplasm. This new HAP-HE@PEI nano-platform is expected to provide a promising system for cell imaging and drug delivery.

## 2. Materials and Methods

### 2.1. Materials

Hydroxyapatite (*Mw*: 502.31 Da, ≥97%, <100 nm particle size (BET)), riboflavin sodium phosphate (*Mw*: 478.33 Da, 93%), Polyethyleneimine (PEI, *Mw*: 1800 Da, 99%) and Doxorubicin hydrochloride (DOX, *Mw*: 579.98, 98%) were purchased from Aladdin (Shanghai, China). All the reagents were used directly without further purification.

### 2.2. Sample Characterization

The microscopic morphologies of the samples were obtained by transmission electron microscopy (TEM, JEOL2100, 200kV, JEOL, Japan). The hydrodynamic size and surface charge were examined by dynamic light scattering (DLS, Zetasizer Nano ZS90, Malvern, Malvern, UK). The crystal structures were investigated using X-ray diffraction (XRD, Rigaku D/MAX 2500V/PC, Rigaku, Japan). The elemental composition and changes were determined by X-ray photoelectron spectroscopy (XPS, Thermo Scientific Escalab 250Xi, Thermo Fisher, USA). Functional groups of the samples were analyzed by Fourier transform infrared spectrometer (FT-IR, Varian 640, Thermo Fisher, USA). The thermogravimetric information was obtained from thermogravimetric analyzer (TGA, TGA55, TA, USA). The fluorescence and UV spectra of the samples were recorded by fluorescence spectrometer (FL, HATICHI, F-7000, Hitachi, Japan) and UV-vis spectrophotometer (Cary 60, Agilent, USA), respectively. Cell imaging was implemented with the high-content imaging system (HCA system, PerkinElmer, Boston, MA, USA).

### 2.3. Preparation of HAP-HE

The HAP-HE was prepared by grafting HE onto the surface of HAP through ligand-exchange reaction [22]. In brief, 254 mg HAP and 72 mg HE were dispersed in 50 mL deionized water and then treated by ultrasonic for 10 min to form a uniform solution. Subsequently, the uniform solution was stirred, and the reaction lasted for 7 h at 60 °C. After that, the as-prepared product HAP-HE was separated by centrifugation (7500 rpm, 5 min), and then purified by centrifugation with deionized water 3 times. At last, samples were freeze-dried to obtain yellow HAP-HE powder.

### 2.4. Preparation of HAP-HE@PEI

HAP-HE@PEI was prepared by wrapping PEI on the surface of HAP-HE. Firstly, 20 mg HAP-HE was dispersed in 25 mL deionized water and stirred to make the dispersion uniform. Subsequently, 2 mL PEI was drawn with a syringe and slowly injected into the dispersion uniform, then stirred at room temperature for 3 h. Finally, the HAP-HE@PEI was obtained by vacuum drying after being purified by centrifugation with deionized water 3 times.

### 2.5. Encapsulation Efficiency and Release Characteristic of HAP-HE@PEI

Doxorubicin hydrochloride (DOX) was used as a test drug to evaluate the encapsulation efficiency of HAP-HE@PEI and to determine its release characteristic under different pH conditions in vitro. In short, 20 mg HAP-HE@PEI and 5 mg DOX were dispersed by ultrasonic in 50 mL phosphate buffer (PBS, pH 7.2), then stirred for 48 h in dark. After 48 h, the solid–liquid separation was achieved by centrifugation (rotational speed: 7500 rpm, 5 min), and the HAP-HE@PEI@DOX complex and supernatant were separated. The absorbance of the supernatant was measured by UV-Vis, and the encapsulation efficiency of HAP-HE@PEI with DOX was calculated according to the standard curve of DOX.

(1) Y=(loaded drug quality)/(Total drug quality)×100 %

According to the formula, the drug loading rate was calculated 83.3%. Then, the release characteristic of HAP-HE@PEI@DOX in PBS with different pH was tested in vitro. The process was as follows: Firstly, 3 mg HAP-HE@PEI@DOX was separately dispersed in the dialysis bag (*Mw*: 7000) with 2 mL PBS (pH 7.2). Then, the dialysis bag was bathed in a release system with 48 mL PBS in different pH (7.2 or 5.4) and stirred gently at 37 °C under dark conditions. At a preset time, 1 mL of liquid was removed from the release system and its absorbance was measured by UV-vis at a wavelength of 488 nm.

### 2.6. Cytotoxicity Evaluation

We used a CCK-8 kit to determine the effects of HAP-HE, HAP-HE@PEI, HAP-HE@PEI@DOX and free DOX on the survival rate of human lung cancer cell line A549. A549 cells were cultured in F-12K medium supplemented with 10% fetal bovine serum (FBS), 1% penicillin and streptomycin and amplified at 37 °C and 5% CO_2_. In detail, A549 cells were inoculated in 96-well plates at a density of 8 × 10^3^ per well and cultured for 24 h. Then the culture medium was removed and 100 μL HAP-HE, HAP-HE@PEI, HAP-HE@PEI@DOX, free DOX and DMEM (blank group) solution with different concentrations (0, 1, 10, 20, 40, 80 μg/mL) was added. After 24 h, the medium was removed and washed three times with PBS, then cells were incubated with 100 μL fresh culture medium containing 10 μL CCK-8 at 37 °C for 1.5 h. The absorbance was measured at 450 nm using a Microplate reader (SpectraMax@ i3).

### 2.7. High Content Cellular Imaging

The NIH-3T3 and A549 cell lines were utilized for high-content cellular imaging. NIH-3T3 cells were cultured in high glucose Dulbecco’s Modified Eagle’s Medium (DMEM) medium supplemented with 10% fetal bovine serum (FBS), 1% penicillin and streptomycin and amplified at 37 °C, 5% CO_2_. A549 cells were cultured as described in 2.6. In detail, NIH-3T3 cells and A549 cells were seeded in 96-well blackboard at a density of 8 × 10^3^ cells per well in 100 μL of the respective media containing 10% fetal bovine serum (FBS). After 24 h of cell attachment, NIH-3T3 and A549 cells were incubated with 12.5–100 μg/mL of HAP-HE or HAP-HE@PEI for preset time (0.5, 1, 3 h). Then, cells were washed 3 times with PBS to remove HAP-HE or HAP-HE@PEI that was not taken up by cells. After that, 50 μL of nucleation reagent Hoechst 33342 was added to each well and incubated in the dark for 30 min. Next, cells were washed three more times with PBS and 100 μL of PBS was re-added to the wells. Plates were then analyzed with HCA System. Three replicate wells were used for each control and test concentrations per well. The obtained fluorescence photos were processed with Image J software, and the results were processed with prism software. The results are presented as the mean ± standard deviation (SD).

### 2.8. HAP-HE@PEI for Cell Imaging of 3D Cultured Cells (Trial)

Macrophage RAW 264.7 cell line was used as a model cell to examine the cell imaging capabilities of HAP-HE@PEI under 3D culture conditions. Bioprinting ink composition: 1 mL 5 wt% GelMA (containing photo-initiator, 0.05 wt% LAP), 0.5 mL 0.02 wt% HAP-HE@PEI. RAW 264.7 cells were collected in the logarithmic growth phase, 1 mL of DMEM medium was added, and we pipetted evenly to obtain a uniformly distributed cell suspension. Then 1 mL of RAW 264.7 cell suspension (approximately 2 × 10^6^ cells/mL) and 1 mL of GelMA containing HAP-HE@PEI were uniformly mixed in a 10 mL centrifuge tube for 30 s at room temperature for 3D-bioprinting (all operations are performed in a clean bench). The 3D inkjet bioprinter (CELLJET Cell Printer, CJ10009, America; dosing distance: 0.5 mm; value opening time: 1000 μs; value closing time: 1000 μs; air pressure: 60 Mpa) drawn 200 microliters of cell-containing bioprinting ink each time, and then printed the preset grid shape in a petri dish (20 × 20 mm) in a continuous printing manner. Subsequently, it was irradiated with UV light for 30 s and then we added high-glucose DMEM medium (containing 10% fetal bovine serum (FBS), 1% penicillin and streptomycin). RAW 264.7 macrophage cells were confined in solidified GelMA hydrogels and cultured at 37 °C, 5% CO_2_ with the medium renewed every 24 h. Fluorescence images were observed and obtained by Leica Microsystems CMS GmbH Ernst-Leitz-Str.17-37 (Leica, Germany).

## 3. Results

### 3.1. Morphological Characteristics of HAP and HAP-HE@PEI

**Figure 1:**
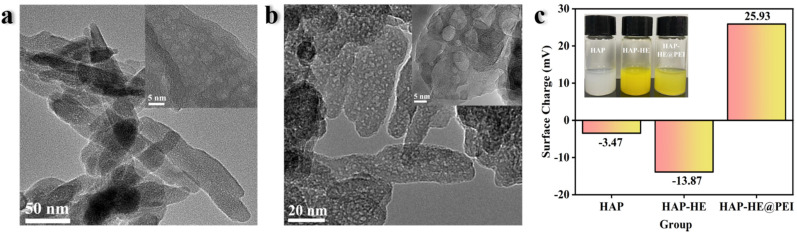
Representative TEM image of (**a**) HAP and (**b**) HAP−HE@PEI; (**c**) surface charge of HAP, HAP−HE and HAP−HE@PEI.

The morphology features of HAP and HAP-HE@PEI composites were characterized by transmission electron microscope (TEM). A single HAP is a spindle-like, with dense and disordered pores of different sizes (Figure 1a). Compared with HAP, the morphology of HAP-HE@PEI did not change, but a high-contrast coating was observed on its surface (Figure 1b). Furthermore, the surface charges of HAP, HAP-HE, HAP-HE@PEI were measured by dynamic light scattering (DLS) technique. According to DLS data, their surface charge was −3.47, −13.87, 25.93 mV, respectively (Figure 1c).

### 3.2. Crystal Structure Comparison of HAP, HAP-HE, HAP-HE@PEI

**Figure 2:**
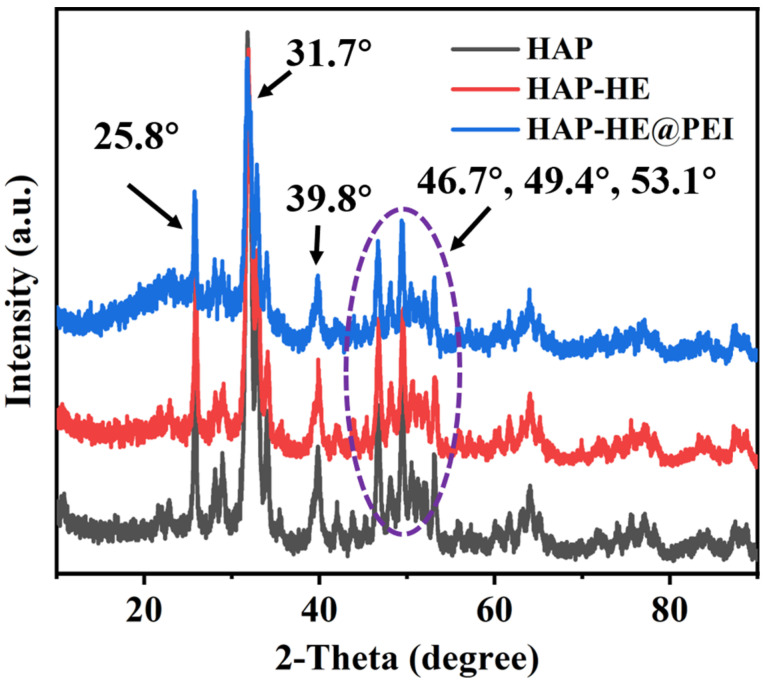
XRD patterns of HAP, HAP−HE and HAP−HE@PEI.

The crystal structures of HAP, HAP-HE, HAP-HE@PEI were characterized by diffraction of X-rays (XRD). In the XRD pattern, HAP exhibits characteristic absorption peaks at 2*θ* of 25.8, 31.7, 39.8, 46.7, 49.4 and 53.1, respectively, and the strongest characteristic peak at 2*θ* of 31.7 (Figure 2). Compared with HAP, the XRD patterns of HAP-HE and HAP-HE@PEI had the same trend and the strongest characteristic peaks at the same position, which implied that both the high stability of HAP and the modification process did not change the crystal structure of HAP.

### 3.3. Element Contents and Changes of HAP, HAP-HE, HAP-HE@PEI

**Figure 3:**
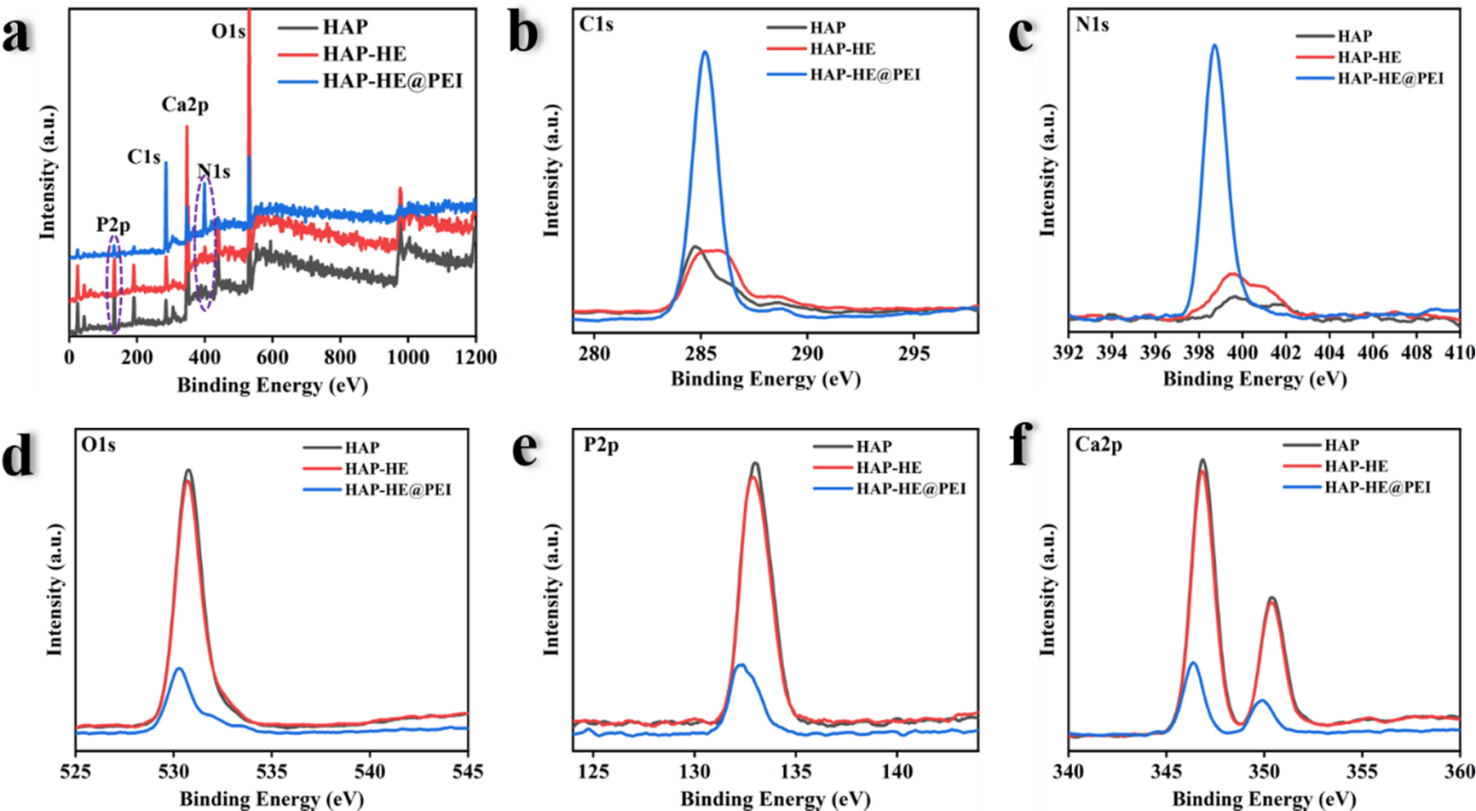
XPS spectra of HAP, HAP−HE and HAP−HE@PEI. (**a**) Survey scan ranging from 0 to 1200 eV, the High-resolution spectra of (**b**) C1s, (**c**) N 1s, (**d**) O 1s, (**e**) P2p and (**f**) Ca2p.

X-ray photoelectron spectroscopy (XPS) was used to analyze the chemical compositions of HAP, HAP-HE and HAP-HE@PEI. The survey of three samples demonstrate that all have the same elemental composition. The XPS spectrum of three samples shows three peaks at 132.0, 286.0, 352.0, 400.0, and 530.6 eV, which are attributed to P2p, C1s, Ca2p, N1s, and O1s, respectively (Figure 3a). The overall atomic percentage of elements present in HAP was carbon (15.78%), nitrogen (4.45%), oxygen (50.67%), calcium (16.26%) and phosphorus (12.84%) (Table S1). After the PEI modified on the surface of HAP-PE, the carbon element in HAP-HE@PEI increased from 15.78% to 51.67%; the nitrogen element increased from 4.45% to 18.18%; the oxygen element decreased from 50.67% to 19.35%, the calcium element decreased from 16.26% to 5.28% and the phosphorus element decreased from 12.84% to 5.21% (Figure 3b–f).

### 3.4. Fourier Transform Infrared Spectroscopy (FT-IR) Spectra of HAP, HAP-HE, HAP-HE@PEI

**Figure 4:**
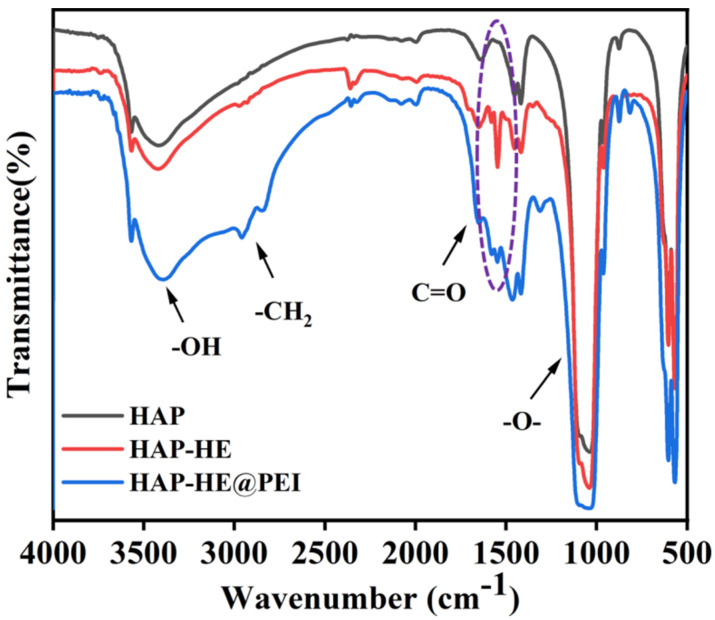
Normalized FT−IR spectra of HAP, HAP−HE and HAP−HE@PEI.

FT-IR spectra was used to further demonstrate the successful surface modification of HAP. At 3400, 1558, 1447, 1045, 848, 598 and 570 cm^−1^, HAP, HAP-HE and HAP-HE@PEI showed the same characteristic absorption peaks as HAP (Figure 4). Compared with HAP, the new characteristic peaks of benzene ring (1644–1454 cm^−1^) and C=O (1702 cm^−1^) emerged in the spectrum of HAP-HE. Moreover, after being modified with PEI, a new peak at 2900 cm^−1^ emerged, which could be attributed to -CH_2_-, and the absorption peak of C=O resulted in a blue-shift phenomenon compared with HAP-HE. In addition, FT-IR was also used to prove that the loading of DOX onto HAP-HE@PEI loaded DOX successfully (Figure S1). For that, DOX has strong characteristic absorption peaks at 3330, 2931, 1729, 1583, 1288 and 804 cm^−1^. Compared with DOX, a new characteristic peak of -O- (1045 cm^−1^) emerged and the intensity at 3330, 2931, 1729, 1583, 1288 and 804 cm^−1^ remarkably decreased in the spectrum of HAP-HE@PEI@DOX.

### 3.5. Thermogravimetric analysis of HAP, HAP-HE and HAP-HE@PEI

**Figure 5:**
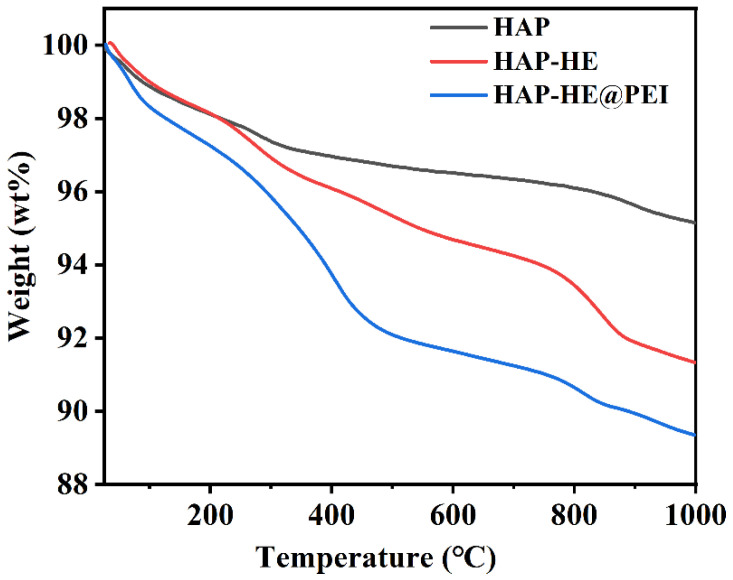
TGA curves of HAP, HAP−HE and HAP−HE@PEI.

The thermogravimetric analyzer (TGA) was used to display the percentage of mass loss of the sample at different temperatures, thereby calculating the mass percentage of the grafted HE and PEI. First, in the range of 25–1000 °C, the mass of samples was all gradually decreased with the increasing test temperature (Figure 5). When the temperature reached 1000 °C, the remaining mass of HAP, HAP-HE and HAP-HE@PEI were 95.16%, 91.33% and 89.35%, respectively. Correspondingly, the weight loss of HAP, HAP-HE and HAP-HE@PEI was approximately 4.84%, 8.67% and 10.65%, respectively. By comparing the remaining masses of HAP and HAP-HE, the mass percentage of HE was approximately 3.83%. Furthermore, by comparing the remaining masses of HAP-HE and HAP-HE@PEI, the mass percentage of PEI was approximately 1.98%.

### 3.6. Fluorescence and UV Properties of HAP, HAP-HE and HAP-HE@PEI

**Figure 6:**
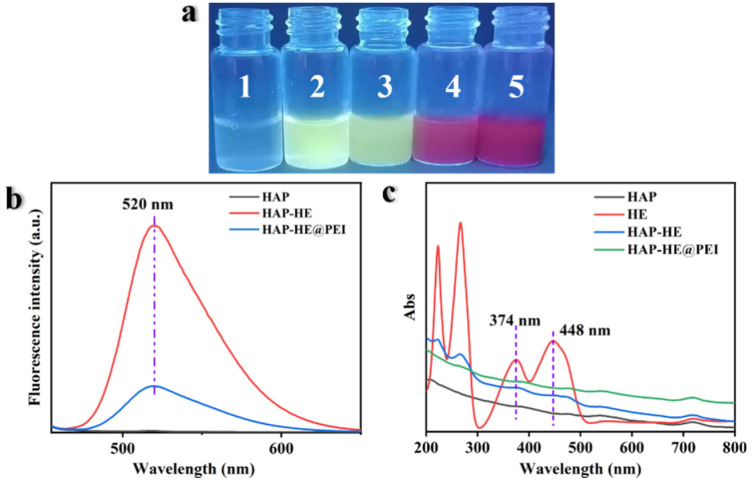
Fluorescence and UV Properties of samples. (**a**) The fluorescence photos of all samples in the UV light box. The numbers 1 to 5 represent HAP, HAP−HE, HAP−HE@PEI, HAP−HE@PEI@DOX and DOX, respectively. (**b**) Fluorescence spectrum (excitation wavelength: 440 nm). (**c**) UV-Vis Spectrum.

Fluorescence spectroscopy (FL) and UV-Vis spectrum was used to analyze the fluorescence and ultraviolet characteristic of HAP, HAP-HE, HAP-HE@PEI, DOX and HAP-HE@PEI@DOX. Photographs of all samples in the UV light box were shown in Figure 6a. HAP does not have fluorescent properties, and it exhibits bright yellow-green fluorescence after grafting HE on the surface. Under 520 nm irradiation, HAP has no characteristic absorption, while HAP-HE shows stronger fluorescence absorption than HAP-HE@PEI (Figure 6b).

From the UV-Vis spectrum, HAP had no absorption whilst HE, HAP-HE and HAP-HE@PEI have absorption peaks at 374 and 448 nm (Figure 6c). Furthermore, under the excitation wavelength of 480 nm, HAP-HE@PEI (520 nm), HAP-HE@PEI@DOX (556 nm, 588 nm) and DOX (556 nm, 588 nm) produced features absorption (Figure S2a). The absorption peak intensity ratios of DOX and HAP-HE@PEI@DOX at 556 and 588 nm decreased from 2.83 (556 nm), 2.35 (588 nm) to 2.28 (556 nm), 1.05 (588 nm). On the other hand, HAP-HE@PEI@DOX and DOX exhibited characteristic absorptions with similar intensities at 480, 540 and 716 nm in the UV-Vis spectrum (Figure S2b).

### 3.7. Cytotoxicity Assay

**Figure 7:**
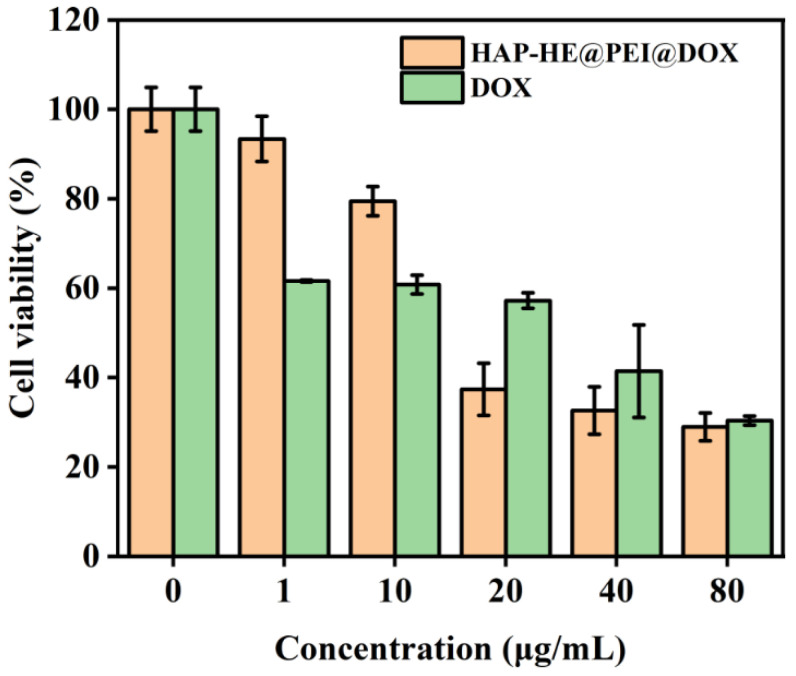
Cytotoxicity assay of HAP−HE@PEI@DOX and free DOX. The cell viability of A549 cells co-incubated with serials of concentrations of HAP−HE@PEI@DOX or DOX.

Here, the A549 lung cancer cells were selected to test the cytotoxicity of HA-HE, HAP-HE@PEI, HAP-HE@PEI@DOX and DOX by CCK-8 assay. After 24 h co-incubation with A549 cells, the cell viability of HAP-HE and HAP-HE@PEI both exceeded 100% in the concentration range of 0–100 μg/mL (Figure S3). Subsequently, the cytotoxicity of HAP-HE@PEI@DOX and DOX was also tested by CCK-8 assay after co-incubation with A549 cells for 24 h. With the increasing concentration of HAP-HE@PEI@DOX and DOX, the cell viability was consistently decreased (Figure 7). When the incubated concentrations of HAP-HE@PEI@DOX and free DOX were higher than 10 μg/mL, HAP-HE@PEI@DOX and free DOX both had cell toxicity.

### 3.8. The Release Characteristic of HAP-HE@PEI@DOX at pH 7.2 and pH 5.4

**Figure 8:**
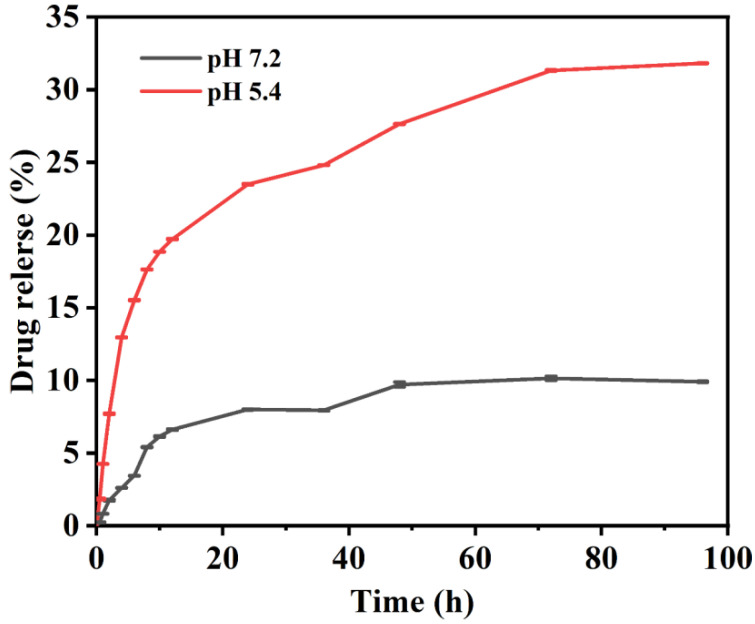
The release behavior of HAP−HE@PEI@DOX to DOX at pH 7.2 and 5.4.

We investigated the loading and release characteristic of DOX on HAP-HE@PEI to evaluate their potential for intracellular drug delivery. Firstly, based on UV-vis spectroscopy, we measured and calculated the loading of DOX on HAP-HE@PEI was 0.293 mg/mg. Afterwards, we further investigated the pH-responsive release of DOX from HAP-HE@PEI@DOX complexes at pH 5.4 and 7.2 (Figure 8). DOX was rapidly released from HAP-HE@PEI@DOX composites at pH 5.4 and 7.2 before the first 20 h (release rate, pH 5.4 > pH 7.2). The release rate of DOX gradually slowed down at pH 5.4 until it reached equilibrium after about 70 h; the release rate of DOX also gradually slowed down at pH 7.2, but reached equilibrium at about 48 h. Obviously, the maximum release of DOX at pH 5.4 (31.83%) was significantly higher than that at pH 7.2 (9.90%), indicating its applications for targeted cancer cell uptake and therapy.

### 3.9. Cell Imaging of 2D Cultured Cells

**Figure 9:**
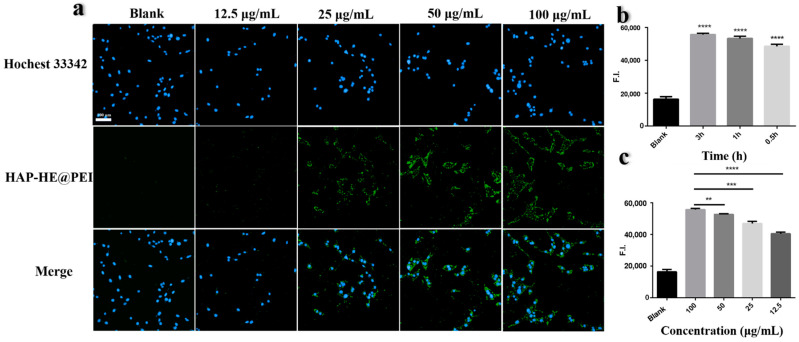
High-content imaging of HAP−HE@PEI. (**a**) Fluorescence images of NIH-3T3 cells co-incubated with different concentrations (12.5, 25, 50, 100 μg/mL) of HAP-HE@PEI for 0.5 h detected by high-content imaging system. (**b**) Fluorescence intensity of HAP-HE@PEI and NIH-3T3 cells incubated with same concentration (100 μg/mL) at different times (0.5, 1, 3 h). Values shown are mean ± S.D., **** *p* < 0.0001, vs. Blank group. (**c**) Fluorescence intensity of HAP-HE@PEI and NIH-3T3 cells incubated with different concentrations (12.5, 25, 50, 100 μg/mL) at the same time (1 h) (HAP-HE@PEI: Ex = 440 nm, Em = 520 nm; Hoechst 33342 for nucleus: Ex = 346 nm, Em = 460 nm). Values shown are mean ± S.D., ** *p* < 0.01, *** *p* < 0.001, **** *p* < 0.0001, vs. 100 μg/mL group, scale bar = 100 μm.

The high-content imaging system was used to obtain and analyze the fluorescence images after co-incubation of HAP-HE@PEI with NIH-3T3 fibroblast cells. The nuclei of NIH-3T3 cells are dotted by Hoechst 33342. Figure 9a showed the fluorescence results: it could be clearly observed that blue fluorescence only exists in the cytoplasm of NIH-3T3 cells. In addition, the fluorescence intensity of HAP-HE@PEI was proportional to its concentration after being taken up by NIH-3T3 cells, and had no significant relationship with the co-incubation time (Figure 9b,c). Among them, HAP-HE@PEI at 100 μg/mL after 3h incubation showed the highest fluorescence intensity. To prove that the surface-cationized HAP-HE@PEI can be taken up by cells more efficiently than HAP-HE, we further used A549 cells to collect their fluorescence images. Under the HAP-HE channel, even with 100 μg/mL and 3h incubation conditions, A549 cells cannot be marked effectively, and the background of green fluorescence was very strong (Figure S4), indicating HAP-HE@PEI has better imaging property than HAP-HE.

### 3.10. Cell Imaging of 3D Cultured Cells

**Figure 10:**
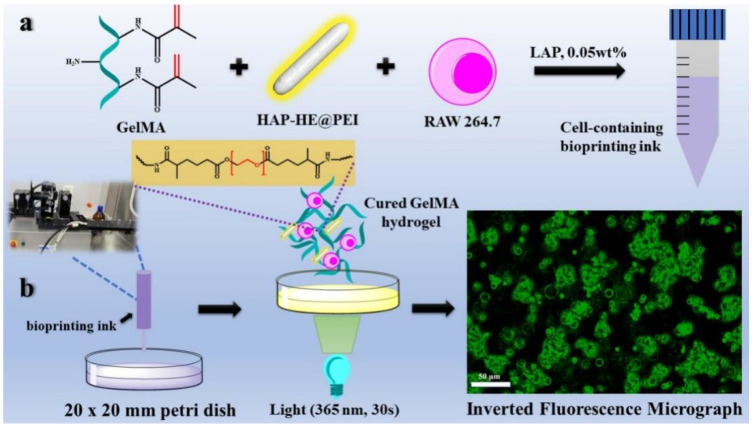
Bioprinting ink preparation and printing for 3D cell imaging process. (**a**) the preparation process of bio-ink (GelMA hydrogel containing 0.02 wt% HAP-HE@PEI). (**b**) 3D cell imaging process including 3D inkjet printing, photocuring, and collection of fluorescent images. (RAW 264.7 cells were grown in bio-ink for 12 h. HAP-HE@PEI: Ex = 440 nm, Em = 520 nm. Scale bar is 50 μm).

We also prepared fluorescent HAP-HE@PEI mixed with GelMA hydrogel as 3D inkjet printing ink, after bio-printing with RAW 264.7 macrophage cells by CELLJET Cell Printer. Figure 10 showed the images observed under an inverted fluorescence microscope (blue filter: Ex = 488 nm), HAP-HE@PEI emitted green fluorescence and RAW 264.7 cells were confined in the GelMA hydrogel. RAW 264.7 cells had multiple cell clusters of different sizes in the hydrogel and grown in longitudinal aggregates, single cell presented a regular circle. These results demonstrated that HAP-HE@PEI is well tolerated by several types of cells.

## 4. Discussion

Good biocompatibility and high imaging efficiency are two necessary prerequisites for nanomaterial cellular imaging [32]. Although Hydroxyapatite (HAP) is widely used for cell imaging due to its good biocompatibility [33,34], its negative charge on the surface has insufficient affinity with the negatively charged cell membrane, resulting in low cell imaging efficiency [35,36]. Therefore, modifying HAP with positively charged compounds to achieve the conversion of HAP surface charge may be one effective strategy to improve the cell affinity. In our study, HAP-HE@PEI with high affinity for cell membranes was successfully prepared by coating the surface of HAP-HE with cationic polymer PEI. Firstly, we confirmed that the morphology of HAP-HE@PEI has no obvious change and the surface become positively charged compared with HAP by TEM and DLS. Then, we confirmed the good biocompatibility of HAP-HE@PEI through the CCK-8 test. In addition, the drug loading and in vitro release characteristic showed that HAP-HE@PEI had great loading capacity for DOX and HAP-HE@PEI@DOX exhibited pH responsiveness. High-content cellular imaging system demonstrated that HAP-HE@PEI had excellent cellular imaging capability. Our results demonstrated that HAP-HE@PEI could be considered as a candidate vehicle for cancer cell imaging and drug delivery at the same time.

Although TEM results suggest that there is no obvious difference in morphology between HAP and HAP in HAP-HE@PEI, more precise data are still needed to prove whether the crystal structure of HAP has changed before and after modification. XRD results showed that the ligand exchange reaction on the surface of HE-grafted HAP and the encapsulation of HAP-HE by PEI were mild, and did not affect the crystal structure of HAP [37].

We also consider the reason for the differential release rate under pH 5.4 and pH 7.2 conditions. The hydrogen ion concentration in PBS solution at pH 5.4 is higher than that in PBS solution at pH 7.2. Due to the dissociation effect of hydrogen ions on HAP-HE@PEI, HAP-HE@PEI@DOX formed a huge concentration difference inside and outside the dialysis bag. Meanwhile, the diffusion behavior of DOX in the dialysis bag might be similarly caused by the concentration difference formed by HAP-HE@PEI@DOX [38].

Finally, to explore the cell imaging potential of HAP-HE@PEI for 3D cultured cells in hydrogels, we chose GelMA as the experimental biomaterial due to its excellent biocompatibility providing cell adhesion sites, supporting cells and allowing nutrients to diffuse [39]. Possible reasons for the different size of cell clusters in the 3D cultured model may be the uneven distribution of cells during the printing process, or the aggregation of cells after printing. In addition, the rigid force that can induce RAW 264.7 cells polarize into other phenotypes exist in the 2D culture environment, but not in the 3D culture environment [40]. The accumulation of green fluorescence in the cell mass was caused by the active uptake of HAP-HE@PEI in GelMA by RAW 264.7 cells. Moreover, the sporadic green circular patterns may be the fluorescence from proliferating cells after uptake of HAP-HE@PEI. Our results show that HAP-HE@PEI has promising imaging potential for RAW 264.7 cells under 3D culture conditions. Based on these findings, we plan to use HAP-HE@PEI with different concentration gradients to eliminate the background fluorescence generated in the 3D environment in future. In addition, as a fluorescent doped material, HAP-HE@PEI may also have application potential for bone repair and bone fluorescent tracing [41]. After reducing the size of HAP (whiskers are about 5 nm), HAP-HE@PEI can also be used as a dopant material in microfluidic chips or as a fluorescent marker to quantify the proliferation of cells in the chip [34,42]. Altogether, combining the advantages of fabrication methods and HAP-HE@PEI, this work thus allows for quick and efficient imaging and drug delivery and is expected to have many in-vitro, ex-vivo and in-vivo applications.

## 5. Conclusions

In summary, we reported a facile and efficient strategy for the fabrication of cationic polymer-functionalized HAP via physical encapsulation. The strategy is based on physical encapsulation (charge attraction), which is simple, fast, efficient and could occur under rather mild conditions (e.g., air, water, catalyst-free, room temperature). After the introduction of PEI, HAP-HE@PEI demonstrated an outstanding cell membrane affinity and good biocompatibility. The HAP-HE@PEI has high encapsulation efficiency and release characteristic with the ability of pH responsiveness. Moreover, high-content imaging analysis shows that HAP-HE@PEI has high biocompatibility, rapid cellular uptake and satisfactory cell imaging ability. Finally, our initial exploration shows that HAP-HE@PEI still has good cell imaging ability under 3D culture conditions. Combining the advantages of fabrication methods and HAP-HE@PEI, this work has implications for biomaterial development aiming methods to improve the efficiency of cellular imaging.

## Data Availability

Not applicable.

## Supplementary Materials

The following supporting information can be downloaded at: https://www.mdpi.com/article/10.3390/bios12060419/s1, Figure S1: Normalized FT-IR spectra of DOX and HAP-HE@PEI@DOX samples; Figure S2: Fluorescence and UV-vis spectra of HAP-HE@PEI@DOX and DOX. (a) FL Spectrum (Excitation wavelength: 480 nm); (b) UV-Vis Spectrum; Figure S3: Determination of cell viability of HAP-HE, HAP-HE@PEI with equal concentration gradient. The cell survival viability of A549 cells co-incubated with gradient mass concentration of HAP-HE and HAP-HE@PEI for 24 h; Figure S4: High-content imaging images of A549 cells co-incubated with 100 μg/mL of HAP-HE for 3h (HAP-HE@PEI: Ex= 440 nm, Em= 520 nm; Hochest 33342 for nucleus: Ex= 346 nm, Em= 460 nm); Table S1: The percentage of each element of HAP, HAP-HE and HAP-HE@PEI.
