# Supplementary material for: Surface-Fabrication of Fluorescent Hydroxyapatite for Cancer Cell Imaging and Bio-Printing Applications
Source: Biosensors (Basel). 2022 Jun 15;12(6):419. doi: 10.3390/bios12060419 (PMC9221440; doi:10.3390/bios12060419)
Supplement: Supplementary file 1 [file biosensors-12-00419-s001.zip › biosensors-1712693-supplementary.pdf]

biosensors

Supplementary

Surface-Fabrication of Fluorescent Hydroxyapatite for Cancer Cell Imaging and Bio-Printing Applications

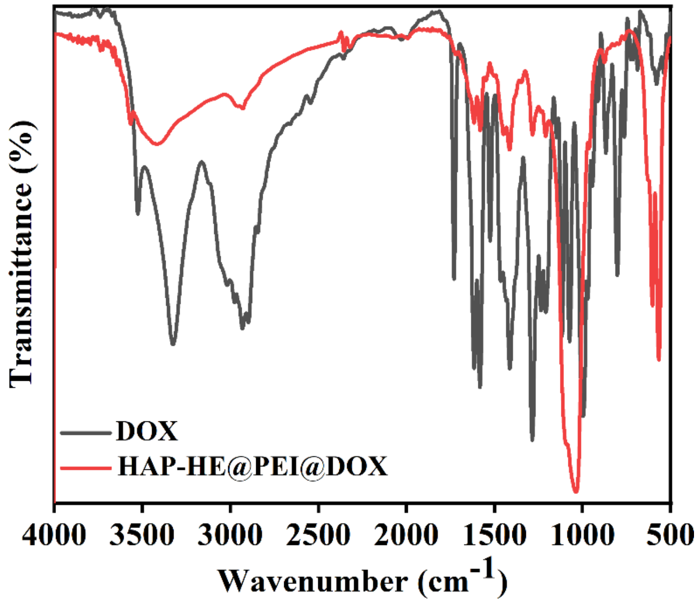

Figure S1. Normalized FT-IR spectra of DOX and HAP-HE@PEI@DOX samples.

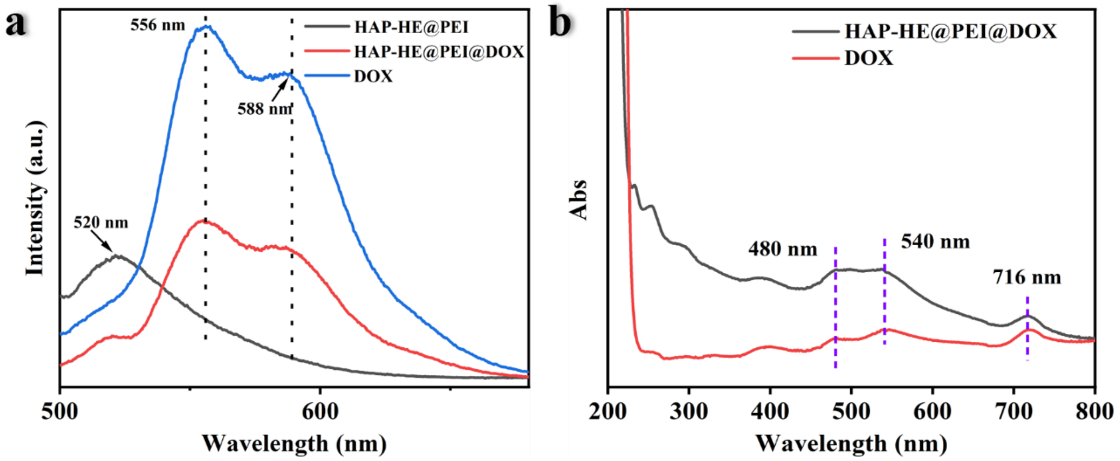

Figure S2. Fluorescence and UV-vis spectra of HAP-HE@PEI@DOX and DOX. (**a**) FL Spectrum (Excitation wavelength: 480 nm); (**b**) UV-Vis Spectrum.

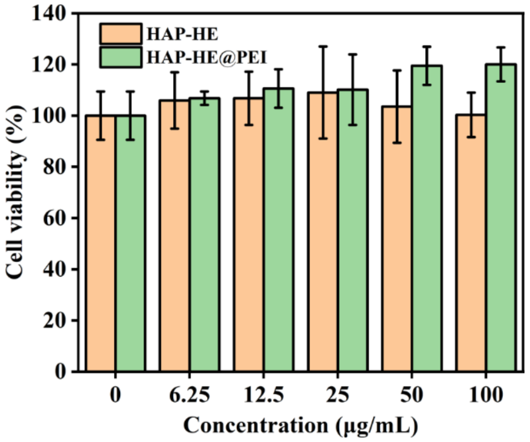

Figure S3. Determination of cell viability of HAP-HE, HAP-HE@PEI with equal concentration gradient. The cell survival viability of A549 cells co-incubated with gradient mass concentration of HAP-HE and HAP-HE@PEI for 24 h.

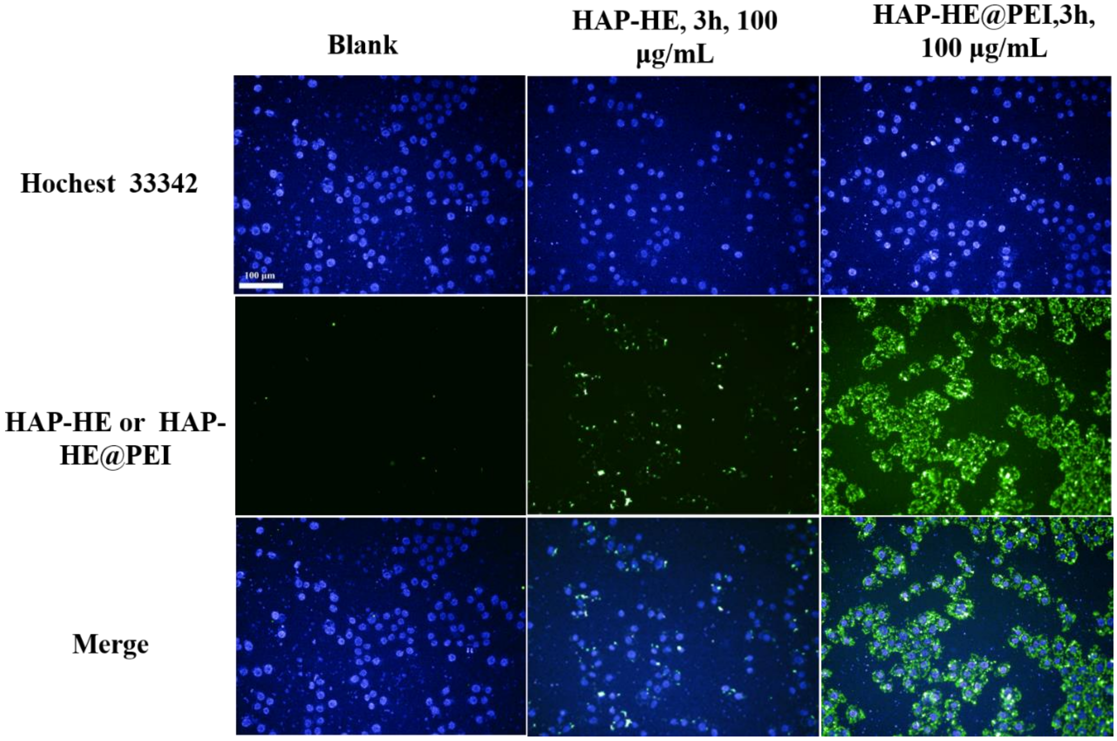

Figure S4. High-content imaging images of A549 cells co-incubated with 100 μg/mL of HAP-HE for 3h (HAP-HE@PEI: Ex= 440 nm, Em= 520 nm; Hochest 33342 for nucleus: Ex= 346 nm, Em= 460 nm).

Table S1. The percentage of each element of HAP, HAP-HE and HAP-HE@PEI.

	C (%)	N (%)	O (%)	P (%)	Ca (%)
HAP	15.78	4.45	50.67	12.84	16.26
HAP-HE	18.18	6.10	48.29	12.13	15.30
HAP-HE@PEI	51.67	18.18	19.35	5.21	5.28
